# Supplementary material for: Phage Digestion of a Bacterial Capsule Imparts Resistance to Two Antibiotic Agents
Source: Microorganisms. 2021 Apr 10;9(4):794. doi: 10.3390/microorganisms9040794 (PMC8069232; doi:10.3390/microorganisms9040794)
Supplement: Supplementary file 1 [file microorganisms-09-00794-s001.zip › microorganisms-1169946-supplementary.docx]

**Supplementary Information for:**

**Phage Digestion of a Bacterial Capsule Imparts Resistance to Two Antibiotic Agents**

Cheng-Hung Luo^1,2*^, Ya-Han Hsu,^1^ Wen-Jui Wu,^3^ Kai-Chih Chang,^3,4*^ Chen-Sheng Yeh^1,2*^

^1^Department of Chemistry, National Cheng Kung University, Tainan 701, Taiwan

^2^Center of Applied Nanomedicine, National Cheng Kung University, Tainan 701, Taiwan

^3^Department of Laboratory Medicine and Biotechnology, Tzu Chi University, Hualien, Taiwan

^4^Department of Laboratory Medicine, Buddhist Tzu Chi General Hospital, Hualien, Taiwan


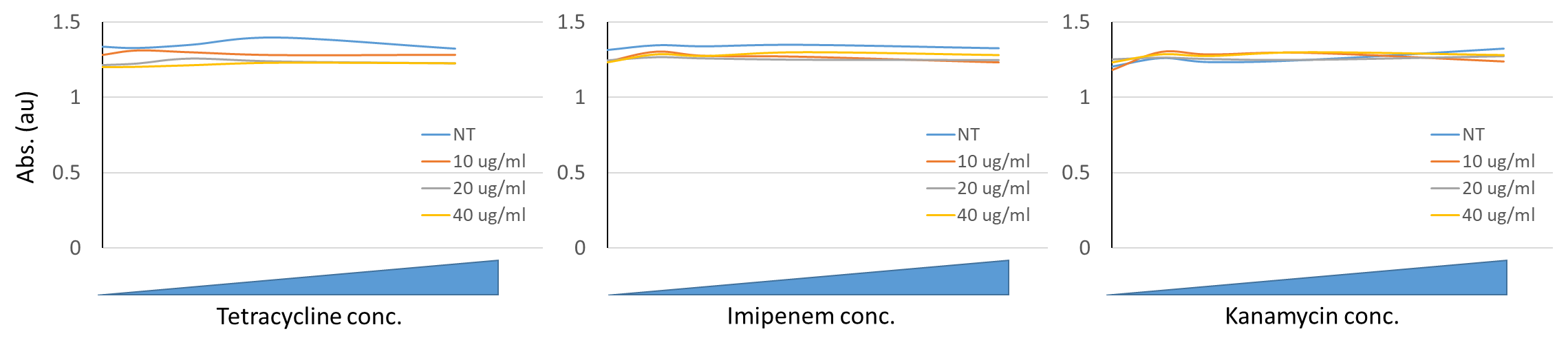


**Figure S1 Bacterial susceptibility to tetracycline, imipenem, and kanamycin in the presence of tail fiber.** Following incubation of MDR *A. baumannii* in the presence of tail fiber protein, we anticipated the attenuated EPS protection by tail fiber treatment may alter bacterial resistance, but no alteration was observed.


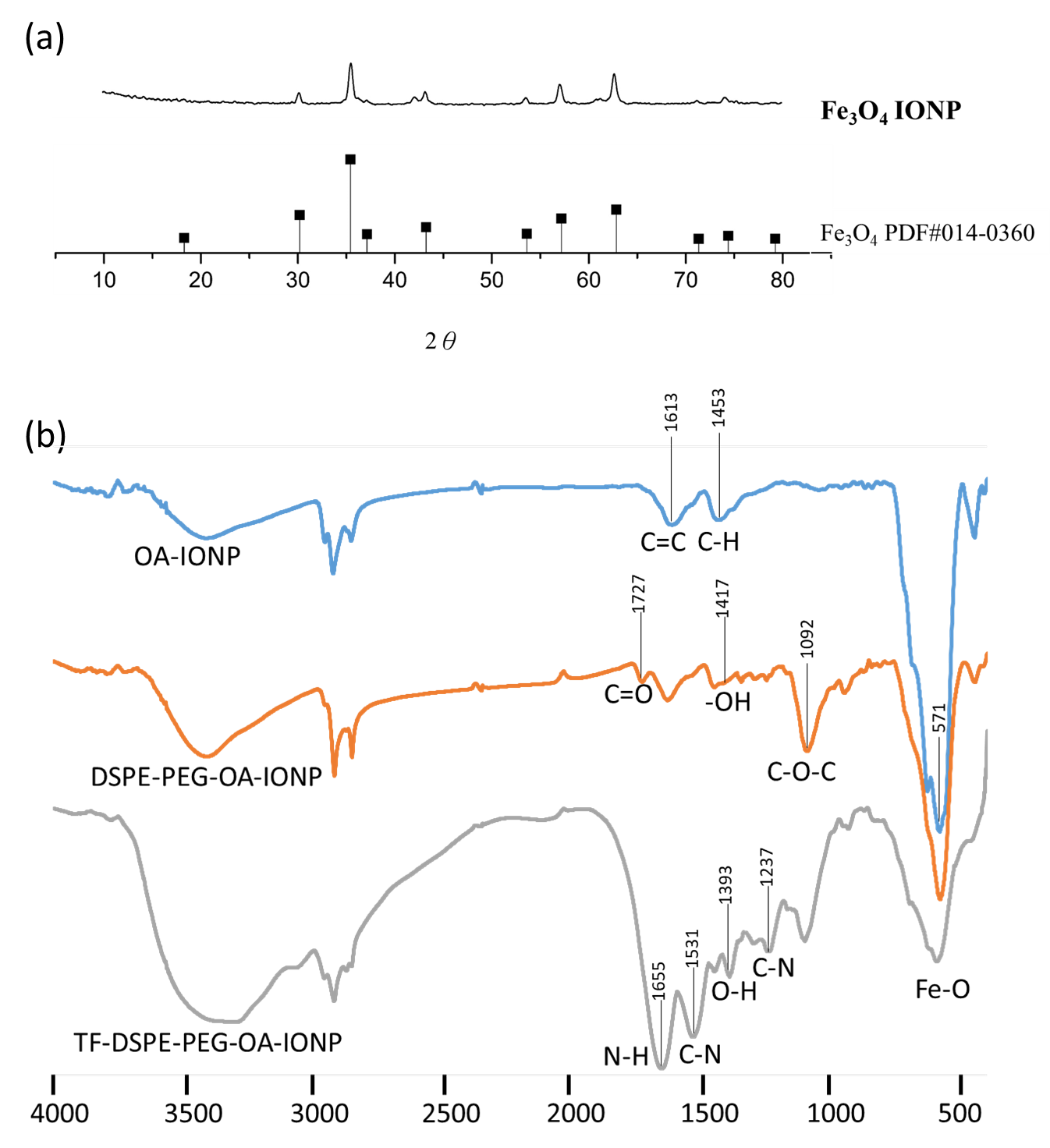


**Figure S2 Identification of Fe_3_O_4_ iron oxide nanoparticles (IONP) and the relevant decoration on IONP.** (a) X-ray powder diffraction (XRD) pattern for the used 22 nm IONP shows peaks corresponding to the reference data. (b) Surface decorations were identified using Fourier-transform infrared spectroscopy (FTIR). The oleic acid (OA) capped IONP shows a Fe-O peak at 571 cm^-1^, along with the typical C-H (1453 cm^-1^) and C=C (1613 cm^-1^) peaks for OA. DSPE-PEG modified IONP shows typical aliphatic ether stretching at 1092 cm^-1^. The tail fiber (TF) anchored IONPs were detected at several peaks at 1237 cm^-1^ (C-N), 1393 cm^-1^ (O-H), 1531 cm^-1^ (C-N), and 1655 cm^-1^ (N-H) in typical protein composition.

**
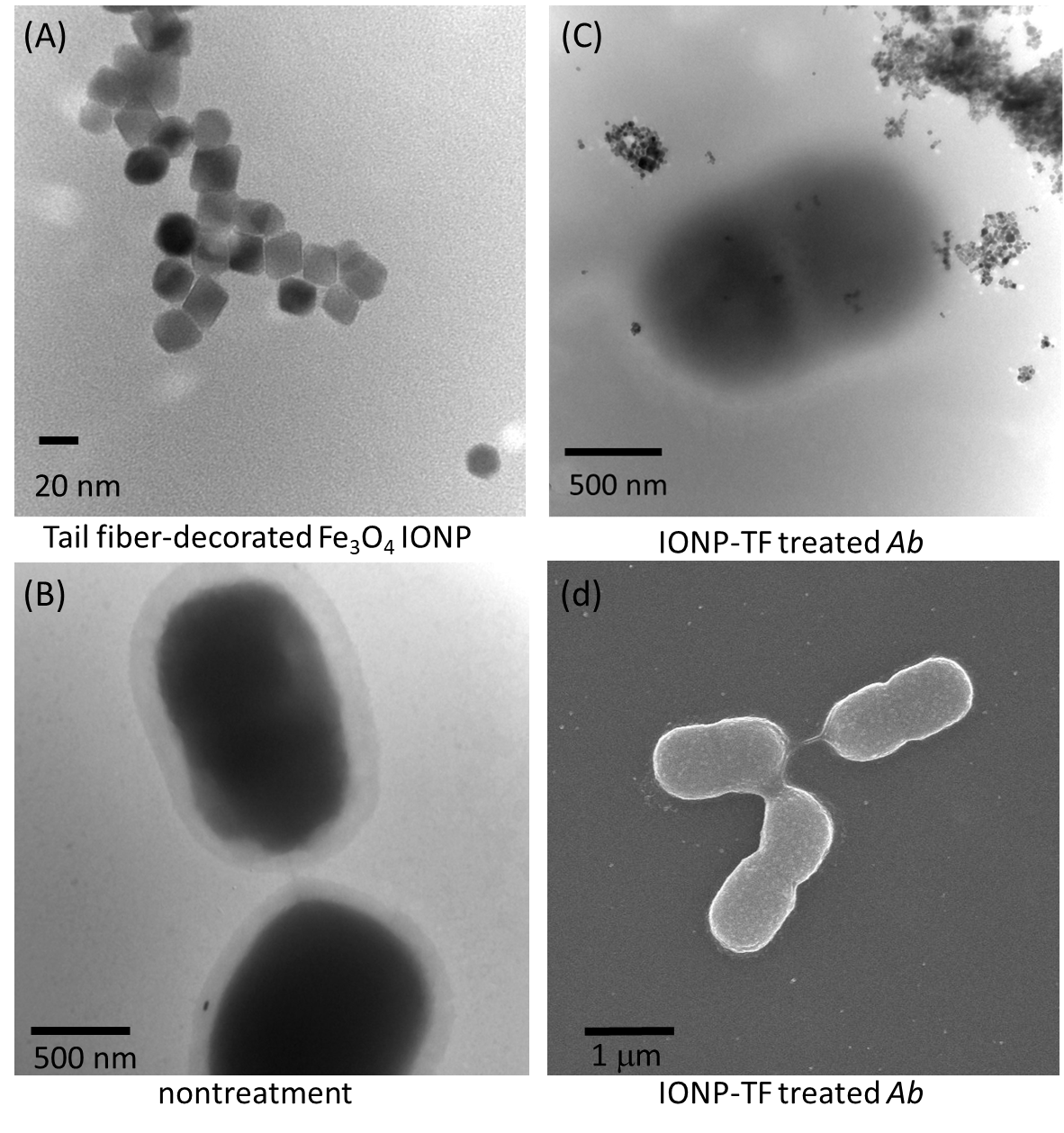
Figure S3 Preparation of tail fiber conjugated iron oxide nanoparticles to treat *A. baumannii*.** (a) TEM image shows the monodispersed tail fiber conjugated iron oxide nanoparticles. (b) *A. baumannii* without treatment shows an intact EPS structure outside the cells. (c) Bacterial cells treated with tail fiber conjugated iron oxide, showing a degraded fuzzy EPS area and iron oxide nanoparticles detached from bacterial cells. (d) Tail fiber conjugated iron oxide treated bacterial cells showed significant morphological change under SEM observation.
